# Translation and cultural adaptation of the CLEFT-Q for use in Colombia, Chile, and Spain

**DOI:** 10.1186/s12955-017-0805-7

**Published:** 2017-11-28

**Authors:** Elena Tsangaris, Karen W. Y. Wong Riff, Federico Vargas, Mirta Palomares Aguilera, Macarena Miranda Alarcón, Asteria Albert Cazalla, Lehana Thabane, Achilleas Thoma, Anne F. Klassen

**Affiliations:** 1 Department of Health Research Methods, Evidence, and Impact, McMaster University, Hamilton, Ontario L8S 4L8 Canada; 2 Division of Plastic and Reconstructive Surgery, Hospital for Sick Children, University of Toronto, Toronto, Ontario M5G 1X8 Canada; 3 Fundación Operación Sonrisa Colombia, Bogotá, 110221 Colombia; 4 Department of Speech and Language Pathology, Fundación Dr. Alfredo Gantz Mann, Santiago, Chile; 5 Department of Plastic Surgery, Hospital Luis Calvo Mackenna, Santiago, Chile; 6 Department of Pediatric Surgery, Hospital Sant Joan de Déu, Barcelona, 08950 Barcelona, Spain; 7 Division of Plastic Surgery, McMaster University, Hamilton, Ontario L8P 3A9 Canada; 8 Department of Pediatrics, McMaster University, Hamilton, Ontario L8S 4K1 Canada

## Abstract

**Background:**

Cleft lip and/or palate (CL/P) is a common congenital craniofacial anomaly that may negatively affect an individual’s appearance, health-related quality of life, or speech. In Spain, Colombia, and Chile the overall prevalence of CL/P ranges from 0.53 to 1.59 cases per 1000 live births. Currently, there is no patient-reported outcome (PRO) instrument that is specific for patients with CL/P. The CLEFT-Q is a new PRO instrument developed to measure outcomes of treatment in patients 8 to 29 years of age with CL/P. The aim of this study was to translate and culturally adapt the CLEFT-Q for use in Colombia, Chile, and Spain.

**Methods:**

The CLEFT-Q was translated from English to 3 Spanish language varieties (Colombian, Chilean, and Spanish (Spain)) and Catalan. Translation and cultural adaptation guidelines set forth by the International Society for Pharmacoeconomics and Outcomes Research were followed.

**Results:**

The field- test version of the CLEFT-Q consisted of 13 scales (total 154 items) measuring appearance, health-related quality of life, and facial function. Forward translations revealed 10 (7%) items that were difficult to translate into Chilean, and back translations identified 34 (22%) and 21 (13%) items whose meaning differed from the English version in at least 1 of the 3 Spanish varieties and Catalan respectively. Twenty-one participants took part in cognitive debriefing interviews. Participants were recruited from plastic surgery centres in Bogotá, Colombia (*n* = 4), Santiago, Chile (*n* = 7), and Barcelona, Spain (*n* = 10). Most participants were males (*n* = 14, 67%) and were diagnosed with CL/P (*n* = 17, 81%). Participants reported difficulty understanding 1 item in the Colombian, 1 item in the Spanish (Spain), and 11 items from the Catalan version. Comparison of the 3 Spanish varieties revealed 61 (40%) of the 154 items whose wording differed across the 3 Spanish versions.

**Conclusion:**

Translation and cultural adaptation processes provided evidence of transferability of the CLEFT-Q scales into 3 Spanish varieties and Catalan, as semantic, idiomatic, experiential, and conceptual equivalence of the items, instructions, and response options were achieved.

**Electronic supplementary material:**

The online version of this article (10.1186/s12955-017-0805-7) contains supplementary material, which is available to authorized users.

## Background

Cleft lip and/or palate (CL/P) is a common craniofacial condition with a global annual prevalence of 7.94 cases per 10,000 live births [1]. In Spain, Chile, and Colombia, the prevalence of CL/P has been reported between 0.53 to 1.59 cases per 1000 live births [2–4].

A diagnosis of CL/P may negatively impact one’s appearance, health-related quality of life (HRQOL), and/or speech [5]. Treatment of CL/P often requires a multidisciplinary team of experts, who follow patients from birth through to adulthood [6]. Patients with CL/P may undergo intensive treatment involving a combination of surgical and non-surgical procedures [7]. Although the goal of treatment is to improve ones’ appearance, psychosocial function, and speech, the measurement of treatment outcomes have traditionally focused on objective clinician- or observer-reported assessments [8–14]. The inclusion of the patient perspective through the use of a specific patient-reported outcome (PRO) instrument for CL/P may increase our understanding of patient concerns, as PRO instruments enable the measurement of a patients’ health concerns [15].

Developing or adapting PRO instruments into Spanish requires careful consideration of the linguistic characteristics of each Spanish variety. Hudson (1996) defines language varieties as *“a set of linguistic items with similar distribution”* [16]. Spanish has been classified as 3 distinct varieties for Spain including, Castilian, Andalusian, and Canary varieties; and 5 distinct varieties for Latin America including, Caribbean, Mexico-Central American, Andean, Rioplatense, and Chilean [17, 18]. Linguistic characteristics of the regional Spanish varieties are distinguished based on differences in phonology (how sounds are used), morphosyntax (the morphological and syntactic properties), and vocabulary [18]. These linguistic features reveal the different categorizations and divisions of the cultural varieties [18]. In a study by García-García et al. (2000), the authors aimed to develop a Spanish (Castillian) versions of the Child Health Assessment Questionnaire (cHAQ), a 30-item disease-specific questionnaire for children with juvenile idiopathic arthritis and other pediatric rheumatic diseases [19, 20]. As part of this study, the authors compared their final Spanish (Castillian) version to the Mexican and Costa Rican translations. Comparison of the Mexican and Spanish (Castillian) versions reveal 24 (80%) items that differed, while the Costa Rican version showed even more semantic differences in 90% of the items when compared to the Spanish (Castillian) [20]. Only 1 question was identical when comparing the Costa Rican and Mexican versions of the questionnaire, with the remaining items indicating some differences [20]. Findings from this study reveal that even among 2 neighboring countries (Mexico and Costa Rica), different adaptations of the cHAQ were needed to meet linguistic and socio-cultural demands, thus supporting the need to develop independent translations that address the linguistic characteristics of Spanish for each Spanish-speaking country or region [20].

In a 2009 report from the United States Food and Drug Administration (FDA) “Guidance for Industry Patient-Reported Outcome Measures: Use in Medical Product Development to Support Labeling Claims” the FDA recommended the provision of “...evidence that the content validity and other measurement properties are adequately similar between all [translated] versions...” [21]. There is an increasing demand for PRO instruments that are available in multiple languages and can be used across different cultures [22]. The CLEFT-Q is a new PRO instrument developed for patients with CL/P to measure the impact of surgery and treatment on ones’ appearance, speech, and HRQOL. To facilitate the involvement of hospitals located in Colombia, Chile, and Spain in an international field- test, a process was required to translate and culturally adapt the CLEFT-Q. Providing translations of the CLEFT-Q that are developed following rigorous methodologies to ensure conceptual and cultural equivalence may enable global benchmarking of outcomes for patients with CL/P who vary by language and culture. The aim of this study was to develop Colombian, Chilean, Spanish (Spain), and Catalan versions of the CLEFT-Q that are conceptually equivalent to the source language version yet are culturally and linguistically appropriate for use in the target country or culture. Translating the CLEFT-Q for use in these countries will facilitate the pooling and comparison of data and will enable assessment of the CLEFT-Q’s transferability, i.e. the degree to which the CLEFT-Q can be transferred to other contexts with other respondents [23, 24]. Best-practice guidelines set forth by the International Society for Pharmacoeconomics and Outcomes Research (ISPOR) for the translation and cultural adaptation of instruments were used [25].

## Methods

### Ethics

This study was approved by the Research Ethics Board at the coordinating centre (Hamilton Integrated Research Ethics Board (HiREB)) and each of the following participating hospitals: Fundación Gantz Hospital del Niño con Fisura in Santiago, Chile; and Hospital Sant Joan de Déu in Barcelona, Spain. For Fundación Operación Sonrisa Colombia and Centro de Atención Multidisciplinaria Gilberto Mariño Contreras in Bogotá, Colombia, the CLEFT-Q study was performed in accordance with the laws set forth by the Ministry of Health Colombia (Resolucion N°008430 De 1993 (4 De Octubre De 1993)). CLEFT-Q study procedures conformed with policies for ethical conduct in research involving humans, and all participants and/or their legal guardians provided written informed consent or assent according to each center’s policy.

### The CLEFT-Q

The CLEFT-Q is a self-report PRO instrument developed for patients with CL/P aged 8 to 29 years, to evaluate the impact of surgery and treatment on a patients’ appearance, speech, and HRQOL [26]. The CLEFT-Q was developed according to guidelines of the United States Food and Drug Administration [21], the Scientific Advisory Committee of the Medical Outcomes Trust [27], and the International Society for Pharmacoeconomics and Outcomes Research [28]. The initial development (Phase I) of the CLEFT-Q involved a literature review [29] followed by qualitative interviews with 138 patients from 6 countries [30]. Results from the literature review was used to develop an initial CLEFT-Q conceptual framework that was refined from the qualitative data and used to inform the development of a set of scales [30]. Revisions to the scales (items, instructions, and response options) were made using feedback from patients during a series of cognitive interviews as well as experts in the field of CL/P [31].

The field- test version of the CLEFT-Q comprised 154 items distributed across 3 domains and 13 concepts as follows: appearance of the face, nose, nostrils, teeth, lips, jaws, cleft scar; HRQOL, i.e., psychological, social, school, and speech-related distress; and facial function i.e., speaking and eating/drinking [31]. The 7 appearance scales ask respondents to answer each item thinking of how their face (or specific area of their face) looks now and respondents are then asked to answer for each item “how much do you like…” using the following 4 response options: “Not at all”, “A little bit”, “Quite a bit”, “Very much” [31]. The HRQOL and facial function scales ask respondents to answer each item in relation to the past week, and in terms of the following frequency response options: “Never”, “Sometimes”, “Often”, “Always” [31]. Mean Flesch–Kincaid (F–K) grade-reading level for the CLEFT-Q items was 1.4 (range, 0–5.2), most of which were below the fifth-grade reading level, with the exception of 2 items [31].

The CLEFT-Q field- test was performed in 30 hospitals across 12 countries [32]. Rasch Measurement Theory (RMT) analysis was used to refine the CLEFT-Q scales and to examine its reliability and validity [32]. The psychometric findings of the final item-reduced CLEFT-Q as well as normative values for age, gender, and cleft type are reported elsewhere [32].

### Selection of translators

For each Spanish variety or Catalan, 3 translators were involved. Two translators whose mother tongue was in 1 of the 3 Spanish varieties or Catalan (target language), and who were fluent in English (source language) were recruited to perform forward translations. Definitions of the key terms used throughout this paper are available in Additional file 1. One additional translator for each Spanish variety or Catalan whose mother tongue was in 1 of the 3 target Spanish varieties or Catalan, and who was fluent in English, was recruited to perform back translations. At least 1 in-country representative (i.e., an individual who lives in the country of the target language) was included as a translator for all Spanish varieties and Catalan [25]. For the Spanish (Spain) and Catalan translations, the same 3 translators performed both translations.

### Selection of study participants for cognitive debriefing interviews

Cognitive debriefing interviews involved participants from each of the participating centers. Eligibility criteria included the following: individuals with CL/P; aged between 8 and 29 years; and could read and understand 1 of the CLEFT-Q target Spanish varieties or Catalan. We aimed to recruit a sample of convenience of 5 participants per Spanish variety and Catalan from the plastic surgery or orthodontics clinics at each center. Sample size for the cognitive debriefing interviews aimed to adhere to the ISPOR recommendations, which is to perform interviews on 5 to 8 participants in the target country [25]. A member of the healthcare team at each center approached potential participants in clinic to invite their participation in the study.

### Translation and cultural adaptation process

**Fig. 1:**
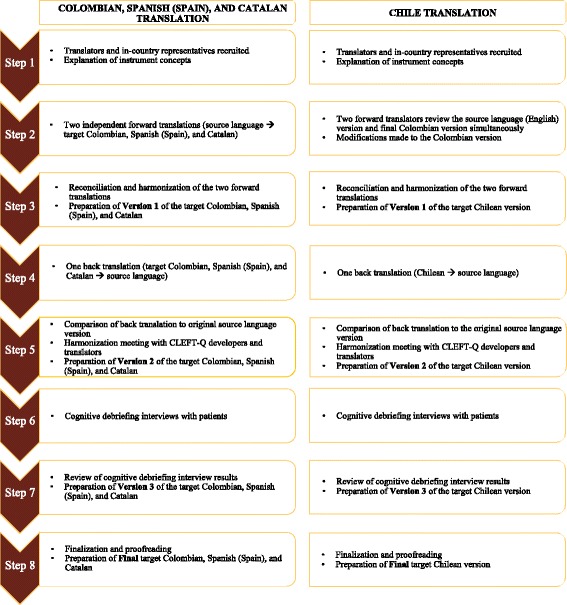
Translation and cultural adaptation steps for the CLEFT-Q

Translation and cultural adaptation of the CLEFT-Q into the 3 Spanish varieties and Catalan took place between November 2014 and June 2016. Best-practice guidelines outlined by ISPOR were used for the translation and cultural adaptation of the CLEFT-Q [25]. The use of these methods ensured the development of high quality reliable translations (Fig. 1).

A member of the CLEFT-Q team (ET) was assigned as the project manager. The project manager reviewed CLEFT-Q concepts with the 2 forward translators for the 4 translations, to ensure that all concepts were clearly understood by the translators as intended by the CLEFT-Q developer. Translations were performed to maintain comparable meaning to the source language version of the items, instructions, and response options [33]. Translators were asked to prepare translations using simple terminology, rather than literal translations, and were encouraged to provide feedback on any words or phrases that were difficult to translate, i.e. due to the items construction, language differences, or any items that may not be culturally acceptable [25]. Difficulty to translate any of the items, instructions, or response options was rated as none (no problems with the translation), minor (some differences in the grammatical or linguistic structure, requiring the item to be expressed in an alternative, yet conceptually equivalent manner), or major (significant differences in the grammatical or linguistic structure such that conceptual equivalence cannot be obtained). The project manager facilitated the translations and analyses for all 4 translations.

Steps for the translation and cultural adaptation the CLEFT-Q were as follows:

Step 1. The project manager oversaw the translation work, trained the translators on the translation procedures to follow to ensure consistency across all translation versions, explained the CLEFT-Q concepts to the 2 individuals performing the forward translation for each of the 4 translations, and reviewed the work after each translation step.

Step 2. Two forward translators whose mother tongue was Colombian, Spanish (Spain), or Catalan, and who were fluent in the source language (English) performed independent forward translations [25]. Translation of the Chilean version entailed 2 forward translators, whose mother tongue was Chilean and who were fluent in the source language (English), who independently reviewed the English version of the CLEFT-Q and used the final Colombian version as a template to suggest modifications that were necessary to achieve semantic, idiomatic, experiential, and conceptual equivalence using acceptable language for the Chilean population [34].

Step 3. After the 2 independent forward translations into the target Spanish variety or language were completed, consensus meetings were held between the 2 forward translator pairs to reconcile their independent forward translations. The Microsoft Excel (2016) worksheet used to reconcile and analyse the forward translation results can be found in Additional file 2. Consensus and reconciliation of the 2 forward translations resulted in Version 1 of each target Spanish variety and Catalan [25].

Step 4. One back translator for each translation, who had not seen the source language (English) version of the CLEFT-Q, translated the target Spanish variety or Catalan Version 1 back into English [25].

Step 5. The project manager compared the back translations for each Spanish variety and Catalan to the source language version to identify discrepancies [25]. More specifically, the project manager compared each item, instruction, and response option in terms of their semantic and idiomatic equivalence (Additional file 1) [34]. The template Excel (2016) worksheet for the analysis of the back translation results can be found in Additional file 3.

Step 6. The project manager, the forward translators, and/or the back translators for each respective Spanish variety and Catalan met to discuss discrepancies between the back translation and the source language version. Challenges in obtaining semantic, idiomatic, experiential, and conceptual equivalence (Additional file 1) of the items, instructions, or response options were further discussed [34]. Items whose meaning was not maintained were re-translated in an iterative manner until an acceptable result was achieved [25]. These steps resulted in Version 2 of the target Spanish varieties and Catalan.

Step 7. For each translation, an in-country representative who was fluent in the target language conducted cognitive debriefing interviews with participants. To maintain consistency in the data collection across all Spanish varieties and Catalan, individuals who performed the cognitive debriefing interviewers were trained by the project manager. Using the ‘think aloud’ approach [35, 36] participants completed the CLEFT-Q while verbalizing each item and what they thought it was asking, which made it possible for the interviewer to identified words and/or phrases that were difficult to understand. For any difficulties identified, the interviewer explained the meaning to the participant, who was then asked to suggest alternative words/phrases to enhance comprehension. This process made it possible to assess the experiential or conceptual equivalence of the CLEFT-Q [25, 34].

Step 8. The project manager and the in-country representative reviewed findings from the cognitive debriefing interviews, which were used to further modify the target Spanish varieties and Catalan versions. This process resulted in Version 3 of the 4 translations [25]. The template Excel worksheet for the analysis of the cognitive debriefing interview results can be found in Additional file 4.

Step 9. The final target Spanish variety and Catalan versions were proofread by 1 of the translators for spelling and grammatical errors. The target Spanish varieties and Catalan versions were included within REDCap (Research Electronic Data Capture, a secure web application for building and managing online surveys and databases [37]) surveys to facilitate the participation of each hospital in the international field- test.

## Results

Translation and cultural adaptation of the CLEFT-Q resulted in the development of 3 equivalent Spanish varieties and a Catalan version for use in Colombia, Chile, and Spain. Translations were developed to be cross-culturally equivalent to the source language version, and the items, instructions, and response options were worded using common language for each Spanish variety and Catalan, so that participants could easily understand them.

Three translators for each Spanish variety and Catalan were recruited to perform the translations. One in-country representative from Colombia and Spain were involved as translators, and 2 from Chile. Individuals performing translations had either no medical background (Colombia, *n* = 1; Chile, n = 1; and Spain, *n* = 2) or were healthcare professionals (Colombia, n = 2; Chile, n = 2; and Spain, n = 1).

### Results from the forward translations of the CLEFT-Q into Colombian, Chilean, Spanish (Spain), and Catalan

**Fig. 2:**
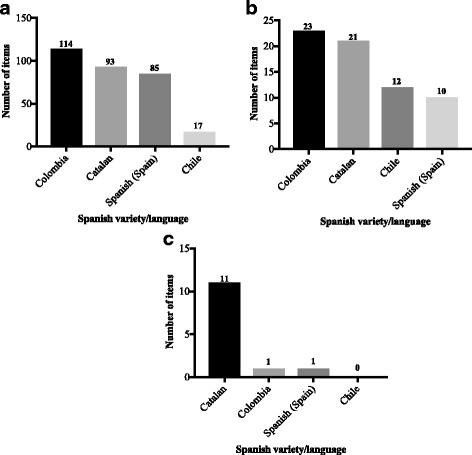
**a**. Total number (*n* = 154) of inconsistent translations of items between the 2 forward translations by Spanish variety/language. **b.** Total number (n = 154) of items whose meaning changed when comparing the back translation to the source language version by Spanish variety/language. **c.** Total number (n = 154) of difficult items for patients during the cognitive debriefing interviews by Spanish variety/language

Reconciliation of the 2 forward translations for each Spanish variety and Catalan revealed some inconsistencies (Fig. 2a). Translation of the CLEFT-Q into Colombian led to a greater number of inconsistencies between the 2 forward translations (*n* = 114, 74%) compared to Chilean (*n* = 17, 11%), Spanish (Spain) (*n* = 85, 55%), and Catalan (*n* = 93, 60%) (Fig. 2a). These inconsistencies were related to the wording or phrasing of the items. For example, in the Colombian version translator 1 translated the item “it’s easy for me to make friends” as “es facil hacer amigos” and translator 2 as “para mi, es facil hacer amigos”. During the consensus and reconciliation meeting, the 2 forward translators agreed that “para mi, es facil hacer amigos” was the best version of the item to depict the source English meaning. Furthermore, of the 154 items in the CLEFT-Q scales, only 10 (7%) items were reported as difficult to translate by the 2 forward translators of the Chilean version, and none were reported as difficult to translate by the forward translators of the Colombian, Spanish (Spain), and Catalan versions. Difficulties expressed by the Chilean translators were considered to be minor. For example, items such as “I feel okay about myself” and “I feel like I fit in” were difficult to translate, as “okay” and “fit in” are phrases not commonly used in Chile. Difficulties with the translations were appropriately resolved after a consensus meeting held between the 2 forward translators and the project manager.

### Results from the back translations of the CLEFT-Q into Colombian, Chilean, Spanish (Spain), and Catalan

Back translation revealed some inconsistencies in the words or phrases of the items for each Spanish variety and Catalan. A total of 144 (94%), 142 (92%), 133 (86%), and 131 (85%) items in the Spanish (Spain), Chilean, Catalan, and Colombian versions respectively had achieved semantic, idiomatic, cultural, and conceptual equivalence. In some instances, when the back translations were compared to the source English version, the meaning of items in the Colombian (*n* = 23, 15%), Catalan (*n* = 21, 14%), Chilean (*n* = 12, 8%), and Spanish (Spain) (*n* = 10, 7%) translations were changed and required re-wording (Fig. 2b). A change in the meaning of an item was more common in the Colombian and Catalan translations. For instance, the item “I stand up for myself” was back translated as “I know how to fend for myself” (Colombian) and “I know how to look after myself” (Catalan). These translations were considered to have a slightly different meaning than the source version and required revision. All discrepancies were resolved after a meeting held between the project manager and the translators for each of the 4 languages.

Semantic equivalence was difficult to achieve for all 4-response option in Colombian, Chilean, and Catalan; however, equivalent translations were later derived. Instructions and response options for the 4 HRQOL scales and 2 facial function scales were easily translated into the 3 Spanish varieties and Catalan. Minor changes to spelling, punctuation, and grammar were required upon final proofreading of the different Spanish varieties and the Catalan versions of the CLEFT-Q.

### Results from cognitive debriefing interviews with participants

Cognitive debriefing interviews involved 21 participants from 3 countries, including Colombia, Chile, and Spain (Table 1). Most participants were male (14, 67%) and had CL/P (17, 81%) (Table 1). For each Spanish variety and Catalan, the number of participants ranged between 4 to 7. In Spain, 5 participants whose mother tongue is Spanish (Spain) and 5 whose mother tongue is Catalan were selected. Catalan-speaking participants expressed the most difficulty interpreting some of the CLEFT-Q items (*n* = 11, 7%) (Fig. 2c). Items such as “how your face looks when you look your best?” and “I feel safe at school (not bullied)” were difficult concepts for some participants. For these items, the words “when you look your best” and “bullied” respectively were identified as difficult to understand. Since no difficulty was expressed for a single item from multiple participants, no changes were made to the translation or to the source version of the CLEFT-Q.

**Table 1:** Characteristics of patients participating in the cognitive debriefing interviews

	Colombia Count (%) *n* = 4	Chile Count (%) *n* = 7	Spanish (Spain) Count (%) *n* = 5	Catalan Count (%) n = 5	Total Count (%) *n* = 21
Age (years)					
8–11	2 (50.0)	0 (0.0)	3 (60.0)	3 (60.0)	8 (38.1)
12–15	0 (0.0)	1 (14.3)	2 (40.0)	1 (20.0)	4 (19.0)
16–19	1 (25.0)	3 (42.9)	0 (0.0)	1 (20.0)	5 (23.8)
20–23	1 (25.0)	2 (28.5)	0 (0.0)	0 (0.0)	3 (14.3)
24–29	0 (0.0)	1 (14.3)	0 (0.0)	0 (0.0)	1 (4.8)
Gender					
Male	3 (75.0)	4 (47.1)	4 (80.0)	3 (60.0)	14 (66.7)
Female	1 (25.0)	3 (42.9)	1 (20.0)	2 (40.0)	7 (33.3)
Cleft type					
Cleft Lip only	0 (0.0)	0 (0.0)	0 (0.0)	1 (20.0)	1 (4.8)
Cleft Palate only	0 (0.0)	0 (0.0)	1 (20.0)	0 (0.0)	1 (4.8)
Cleft Lip and Palate	4 (100)	6 (85.7)	4 (80.0)	3 (60.0)	17 (80.9)
Cleft Lip and Alveolus	0 (0.0)	1 (14.3)	0 (0.0)	1 (20.0)	2 (9.5)

### Example results from the Chilean translation and cultural adaptation process of the CLEFT-Q

Table 2 provides an example of the changes made throughout the process of translating the Chilean version of the CLEFT-Q. Reconciliation of the 2 forward translations revealed 17 (11%) items that were translated differently by the 2 forward translators. Among these items, 10 (7%) consisted of words or phrases that were considered difficult to translate (Table 2). For example, the item “how your cleft lip scar looks from far away?” was translated as “how do you see the lip scar from afar?” by translator 1 and “how does the scar on your lips look from afar?” by translator 2. The translation of translator 1 was considered to be the most appropriate to maintain the meaning of the source item and was retained in Version 1. Back translation revealed 135 (87%) items whose wording differed slightly from the source version, among which the meaning of 12 (8%) items was considered different (Table 2). Items whose meaning was changed from the source language version were re-translated until a satisfactory result was achieved. Cognitive debriefing interviews conducted with 7 Chilean-speaking participants reveal no difficulty understanding the words or phrases of the items.

**Table 2:** Example of the changes made throughout the translation of the Chilean version of the CLEFT-Q

Scale	Item	Difficult to Translate (specific word or phrase)	Solution	Changed meaning post back translation (specific word or phrase)	Solution	Difficult for patients to understand or interpret (specific word or phrase)	Solution
Face	…how your face looks when you look your best?	Yes (when you look your best)	Excluded “when you look your best” from Chilean version	Yes	Used equivalent Chilean phrase for “when you look great”	No	n/a
Face	…how your face looks when you laugh?	No	n/a	Yes (laugh)	Used equivalent Chilean phrase for “laugh out loud”	No	n/a
Nose	...how the bridge of your nose looks (the part of the nose where glasses sit)?	Yes (bridge)	Used equivalent Chilean phrase for “top of the nose where the glasses are placed”	No	n/a	No	n/a
Nose	…how wide your nose looks at the bottom (from side to side)?	Yes (at the bottom)	Excluded “at the bottom” from Chilean version	No	n/a	No	n/a
Nose	…the shape of your nose (how flat or raised up it is)?	Yes (flat or raised up)	Excluded “(how flat or raised up it is)” from Chilean version	No	n/a	No	n/a
Nostrils	…the width of your nostrils (from side to side)?	Yes (from side to side)	Excluded “(from side to side)” from Chilean version	No	n/a	No	n/a
Lips	…how much you can move your lips (like to whistle or kiss)?	No	n/a	Yes (how much you can move)	Used equivalent Chilean phrase for “how your lips move”	No	n/a
Lips	…how your lips look when you laugh?	No	n/a	Yes (laugh)	Used equivalent Chilean phrase for “laugh out loud”	No	n/a
Cleft Lip Scar	…how your cleft lip scar feels when you touch it (smooth or bumpy)?	Yes (smooth or bumpy)	Excluded “(smooth or bumpy)” from Chilean version	No	n/a	No	n/a
Cleft Lip Scar	…how your cleft lip scar looks in the mirror?	No	n/a	Yes (how your)	Used equivalent Chilean phrase for “what does the scar on the lip look like in the mirror”	No	n/a
Jaws	…how far your bottom jaw comes out compared to your top jaw?	No	n/a	Yes (compared to your top jaw)	Used equivalent Chilean phrase for “compared to your top jaw”	No	n/a
Psychological	I feel okay about myself.	Yes (okay)	Used equivalent Chilean word for “comfortable”	No	n/a	No	n/a
Psychological	I feel like I fit in.	No	n/a	Yes (fit in)	Used equivalent Chilean phrase for “I belong”	No	n/a
School	Other students listen to me when I talk.	No	n/a	Yes (listen to me)	Used equivalent Chilean phrase for “listen to me”	No	n/a
School	I fit in at school.	No	n/a	Yes (fit in)	Used equivalent Chilean phrase for “I feel part of my school”	No	n/a
Social	I have fun with friends.	No	n/a	Yes (have fun)	Used equivalent Chilean phrase for “have fun”	No	n/a
Social	I feel like I fit in with people.	Yes (fit in)	Used equivalent Chilean phrase for “I feel that I integrate well with people”	No	n/a	No	n/a
Social	It’s okay when people look at my face.	Yes (okay)	Used equivalent Chilean phrase for “it is fine”	No	n/a	No	n/a
Social	It’s okay if people ask me about my face.	Yes (okay)	Used equivalent Chilean phrase for “it is fine”	No	n/a	No	n/a
Speech Function	I need to try hard to speak well.	No	n/a	Yes (I need to try hard)	Used equivalent Chilean phrase for “need to make an effort”	No	n/a
Eating/Drinking	I have trouble biting into some foods.	No	n/a	Yes (I have trouble)	Used equivalent Chilean phrase for “have trouble”	No	n/a
Eating/Drinking	I have to avoid eating certain foods.	No	n/a	Yes (have to)	Used equivalent Chilean phrase for “have to”	No	n/a

n/a = not applicable

### Comparison of item wording between the final Colombian, Chilean, and Spanish (Spain) versions of the CLEFT-Q

A total of 61 (40%) items differed across the 3 Spanish varieties. Comparison of the 2 South American versions (Colombian and Chilean) revealed that although the meaning of the items was maintained, the item construction or wording of 84 (55%) items differed. Similarly, 84 (55%) items differed between the Colombian and Spanish (Spain) versions, while comparison of the Chilean and Spanish (Spain) version revealed that 98 (64%) items differed in there item construction or wording. Table 3 outlines the total number of differences of the CLEFT-Q item wording by domain between the 3 Spanish varieties. Interestingly, more differences between items in the appearance scales were identified compared to items in the HRQOL and facial function scales (Table 3). An example of the differences in the items of the cleft lip scar scale between the 3 Spanish varieties can be found in Table 4.

**Table 3:** Total number of differences of the CLEFT-Q items between the 3 Spanish varieties

	Appearance scales Count (%) *N* = 79 items	Health-related Quality of Life scales Count (%) *N* = 51 items	Facial Function scales Count (%) *N* = 24 items	Total Count (%) N = 154 items
Colombia versus Chile	59 (74.7)	17 (33.3)	8 (33.3)	84 (54.6)
Colombia versus Spanish (Spain)	56 (70.9)	18 (35.3)	10 (41.7)	84 (54.6)
Chile versus Spanish (Spain)	62 (78.5)	25 (49.0)	11 (45.8)	98 (63.6)

**Table 4:** Example differences of items from the cleft lip scar scale between the 3 Spanish varieties

Original English item	Colombian translation	Chilean translation	Spanish (Spain) translation
…how your cleft lip scar looks from far away?	…cómo ves la cicatriz del labio hendido desde lejos?	…cómo se ve la cicatriz de tu fisura labial desde lejos?	…tu cicatriz de fisura labial desde lejos?
…how much your cleft lip scar has faded over time?	…cuánto se ha borrado la cicatriz con el tiempo?	…cuánto se ha borrado la cicatriz del labio fisurado con el tiempo?	…cuánto se ha borrado tu cicatriz con el tiempo?
…how much the colour of your cleft lip scar matches your skin colour?	…lo parecido del color de la cicatriz con el color de tu piel de alrededor?	…lo parecido del color de la cicatriz del labio fisurado con el color de tu piel de alrededor?	…lo que se parece el color de tu cicatriz con el color de tu piel de alrededor?
…how your cleft lip scar feels when you touch it (smooth or bumpy)?	…cómo sientes la cicatriz del labio hendido al tocarla?	…cómo se siente la cicatriz de tu fisura labial al tocarla?	…cómo sientes tu cicatriz de fisura labial al tocarla?
…the colour of your cleft lip scar?	…el color de la cicatriz de tu labio hendido?	…el color de la cicatriz del labio fisurado?	…el color de tu cicatriz de fisura labial?
…how your cleft lip scar looks in the mirror?	…la cicatriz de tu labio hendido en el espejo?	…cómo se ve la cicatriz del labio fisurado en el espejo?	…tu cicatriz de fisura labial en el espejo?
…how your cleft lip scar looks in photos?	…la cicatriz de tu labio hendido en las fotografías?	…cómo se ve la cicatriz del labio fisurado en las fotografías?	…tu cicatriz de fisura labial en las fotografías?
…the width of your cleft lip scar?	…el ancho de la cicatriz de tu labio hendido?	…el ancho la cicatriz del labio fisurado?	…el ancho de tu cicatriz de fisura labial?
…how your cleft lip scar looks when you smile?	…la cicatriz de tu labio hendido cuando sonríes?	…cómo se ve la cicatriz del labio fisurado cuando sonríes?	…tu cicatriz de fisura labial cuando sonríes?
…the size of your cleft lip scar?	…el tamaño de la cicatriz de tu labio hendido?	…el tamaño de la cicatriz del labio fisurado?	…el tamaño de tu cicatriz de fisura labial?
…how your cleft lip scar looks up close?	…cómo ves la cicatriz del labio hendido desde cerca?	…cómo se ve la cicatriz del labio fisurado desde cerca?	…tu cicatriz de fisura labial de cerca?
…the shape of your cleft lip scar?	…la forma de la cicatriz de tu labio hendido?	…la forma de la cicatriz del labio fisurado?	…la forma de tu cicatriz de fisura labial?

## Discussion

Our team has developed 4 conceptually equivalent translations of the CLEFT-Q prior to commencing our international field-test. Performing advanced translations was essential to gaining input from different cultural and linguistic backgrounds before finalizing the source questionnaire for cross-cultural implementation [38]. To achieve maximum equivalence of items, instructions, and response options, it is crucial that the process of cross-cultural translation of a PRO instrument follows a valid and scientifically sound methodology [25]. Achieving cross-cultural equivalence of the CLEFT-Q was crucial to enable its use in multiple Spanish-speaking countries, and to facilitate their participation in the international field- test study [39].

Comparison of the 2 forward translations for the Chilean version revealed fewer inconsistencies compared with the Colombian, Spanish (Spain), and Catalan versions. These fewer inconsistencies may be attributed to the fact that the Colombian version was used as a template, alongside the English version, to develop the Chilean translation. Analysis of the back translations revealed some discrepancies in the items’ meaning when compared to the source language version. However, all of the items whose meaning was changed during the translation process were easily modified for the final version to reflect the meaning of the source language version. Finally, comparisons of the 3 Spanish varieties reveal substantial differences between each version. These findings, which highlight the importance of having separate translations for different Spanish countries, are consistent with results from the study by García-García et al. (2000) who identified that over 80% of the items differed between the Mexican, Costa Rican, and Spanish (Spain) versions of the cHAQ [20].

It was important to develop conceptually equivalent, rather than literal translations for each Spanish variety and Catalan [25]. To achieve conceptual equivalence, initial explanations of the items, instructions, and response options, as well as frequent discussion between the translators, project manager, and the instrument developers was necessary. Despite the significant grammatical differences between English (ie. a Germanic language) and Spanish (ie. a Romance language), no major challenges arose during the translation and cultural adaptation of the CLEFT-Q into the multiple Spanish varieties and Catalan due to the easy application of the ISPOR guidelines, as well as the simple and objective organization and wording of the CLEFT-Q items, instructions, and response options. Translation and cultural adaptation of the CLEFT-Q allowed us to make critical changes to the items, instructions, or response options prior to launching our international field- test. Input from participants with CL/P was essential to ensure that the CLEFT-Q was easily understood and applicable to the target populations, and contributed to some improvements to the initial translations. Analysis of semantic, idiomatic, cultural, and conceptual equivalence confirmed that the CLEFT-Q constructs are appropriate and are equally valid for the target Spanish variety and Catalan.

Two different versions of the CLEFT-Q were prepared for use in Spain, Spanish and Catalan. Despite the fact that Spanish and Catalan are spoken within the same country and are both Romance languages, comparison of the 2 versions revealed no overlap between the items, instructions, or response options of the CLEFT-Q. Therefore, translation of the CLEFT-Q into both languages was necessary to facilitate the use of the CLEFT-Q in Spain, particularly Barcelona. Also, using the Colombian version as a template to develop the Chilean version proved to be effective, and reduced the time needed to prepare the Chilean translation. Future translations of the CLEFT-Q into other Spanish varieties from neighboring countries may warrant the use of either the Colombian, Chilean, or Spanish (Spain) versions as a template to prepare appropriate translations for their populations. However, translation and cultural adaptation of the CLEFT-Q into other languages, including other Romance languages, would require their own translations using the source English version of the CLEFT-Q.

Since the goal for our international field- test (Phase II) was to include multiple countries [32], our team decided it was critical to perform the translation and cultural adaptation work prior to validating the scales. This approach enabled us to ensure that the content of the CLEFT-Q resonated well with participants with CL/P who vary by country and language. Therefore, an important strength of our study is the inclusion of the translation and cultural adaptation procedures during Phase I of the CLEFT-Q development. Furthermore, the inclusion of an international sample of participants for the cognitive debriefing interviews simultaneously proved to be advantageous for confirming the transferability of the CLEFT-Q. Finally, consistent methodology used throughout the translation and cultural adaptation process was maintained by having a member of the CLEFT-Q team (ET) train all the translators and cognitive debriefing interviewers on the procedures.

A potential limitation of the present study was that the 3 translators used to perform the translations of each Spanish variety and Catalan were not professional translators. Also, each translator who performed the back translations had a mother tongue in the respective Spanish variety or Catalan, and were fluent in English. ISPOR recommendations are to use professional translators, and for the back translations to be performed by someone fluent in the target language with their mother tongue in English. However, we think it is unlikely these deviations from ISPOR guidelines had any impact on the quality of the final translations. Another limitation is our use of a convenience sample of participants for the cognitive debriefing interviews. Given that a small number of participants are required for this final step of the ISPOR translation and cultural adaptation process, it is not possible to ensure a representative sample is chosen. For example, our sample mostly included participants with CL/P as opposed to other cleft types. However, this difference reflects the distribution of cleft types in the literature [40]. Lastly; only 4 participants were included in the cognitive debriefing interviews for the Colombian translations, which is 1 fewer than the ISPOR recommendations. Since multiple translations were completed simultaneously, the feedback obtained from a large sample of Spanish participants completing the other translations, we feel it unlikely that the addition of 1 more participant would have changed the final Colombian version.

## Conclusion

Translation and cultural adaptation processes provided evidence of transferability for the CLEFT-Q scales into 3 Spanish varieties and Catalan, as semantic, idiomatic, experiential, and conceptual equivalence of the items, instructions, and response options was achieved. Upon completion of the CLEFT-Q development the scales will be available for use in clinical practice, research, and benchmarking of outcomes internationally. Methods for the translation and cultural adaptation of the CLEFT-Q described here can be used to assess the quality and validity of our translation, and to inform new translation of the CLEFT-Q as well as other PRO instruments.

## Additional files

Additional file 1: Key definitions. (DOCX 86 kb)

Additional file 2: Template data collection and analysis form for forward translation. (DOCX 65 kb)

Additional file 3: Template data collection and analysis form for back translation. (DOCX 67 kb)

Additional file 4: Template data collection and analysis form for cognitive debriefing interviews. (DOCX 65 kb)

## Abbreviations

cHAQ: Child Health Assessment Questionnaire
CL/P: Cleft lip and/or palate (CL/P)
FDA: United States Food and Drug Administration
F–K: Flesch–Kincaid
HiREB: Hamilton Integrated Research Ethics Board
HRQOL: Health-related quality of life
ISPOR: International Society for Pharmacoeconomics and Outcomes Research
PRO: Patient-reported outcome
REDCap: Research Electronic Data Capture
RMT: Rasch Measurement Theory
