# Supplementary material for: Translation and cultural adaptation of the CLEFT-Q for use in Colombia, Chile, and Spain
Source: Health Qual Life Outcomes. 2017 Nov 28;15:228. doi: 10.1186/s12955-017-0805-7 (PMC5704495; doi:10.1186/s12955-017-0805-7)
Supplement: Supplementary file 1 — Key definitions. (DOCX 86 kb) [file 12955_2017_805_MOESM1_ESM.docx]

**Supplementary file 1.** Key definitions

| **Term** | **Definition** |
| --- | --- |
| Source language | The language in which the instrument was developed^41^ |
| Target Spanish variety/language | The Spanish variety/language in which the source language will be translated and culturally adapted^41^ |
| Forward translation | The process of translating the source language into the target Spanish variety/language, which requires 2 translators who are native speakers of the target Spanish variety/language, and bilingual in the source language^25,41^ |
| Back translation | The process of translating the target Spanish variety/language version back into the source language, which requires one translator who is a native speaker of the source language, and bilingual in the target Spanish variety/language^25,41^ |
| Cognitive debriefing interviews | The process of testing the target Spanish variety/language version on patients from the target population to identify difficulties with comprehension. Face-to-face interviews are conducted to test how individuals understand and interpret the instrument items instructions and response options^25,41^ |
| Semantic equivalence | Similarity in the meaning of words^34^ |
| Idiomatic equivalence | Similarity in the translation of idioms and colloquial terminology^34^ |
| Experiential equivalence | The content of the instrument is culturally equivalent^34^ |
| Conceptual equivalence | Concept validity in relation to experiences of individuals in the target culture^34^ |
