# Supplementary material for: Translation and cultural adaptation of the CLEFT-Q for use in Colombia, Chile, and Spain
Source: Health Qual Life Outcomes. 2017 Nov 28;15:228. doi: 10.1186/s12955-017-0805-7 (PMC5704495; doi:10.1186/s12955-017-0805-7)
Supplement: Supplementary file 2 — Template data collection and analysis form for forward translation. (DOCX 65 kb) [file 12955_2017_805_MOESM2_ESM.docx]

**Supplementary file 2.** Template data collection and analysis form for forward translation

| Translator #1: ___*name*___ | | | | | | |
| --- | --- | --- | --- | --- | --- | --- |
| Translator #2: ___*name*___ | | | | | | |
| CLEFT-Q scale | Source language CLEFT-Q item | Translation 1 | Translation 2 | Inconsistency between translations? Y/N | What was the inconsistency? | What was the solution? |
|  |  |  |  |  |  |  |
|  |  |  |  |  |  |  |
|  |  |  |  |  |  |  |
|  |  |  |  |  |  |  |
|  |  |  |  |  |  |  |
|  |  |  |  |  |  |  |
|  |  |  |  |  |  |  |
|  |  |  |  |  |  |  |
|  |  |  |  |  |  |  |
|  |  |  |  |  |  |  |
|  |  |  |  |  |  |  |
|  |  |  |  |  |  |  |
|  |  |  |  |  |  |  |
|  |  |  |  |  |  |  |
