# Supplementary material for: Translation and cultural adaptation of the CLEFT-Q for use in Colombia, Chile, and Spain
Source: Health Qual Life Outcomes. 2017 Nov 28;15:228. doi: 10.1186/s12955-017-0805-7 (PMC5704495; doi:10.1186/s12955-017-0805-7)
Supplement: Supplementary file 3 — Template data collection and analysis form for back translation. (DOCX 67 kb) [file 12955_2017_805_MOESM3_ESM.docx]

**Supplementary file 3.** Template data collection and analysis form for back translation

| CLEFT-Q scale | Source language CLEFT-Q item | Back translated item | Wording discrepancy? Y/N | Change of item meaning? Y/N | Comments | Translation changed?  Y/N | What was the change? |
| --- | --- | --- | --- | --- | --- | --- | --- |
|  |  |  |  |  |  |  |  |
|  |  |  |  |  |  |  |  |
|  |  |  |  |  |  |  |  |
|  |  |  |  |  |  |  |  |
|  |  |  |  |  |  |  |  |
|  |  |  |  |  |  |  |  |
|  |  |  |  |  |  |  |  |
|  |  |  |  |  |  |  |  |
|  |  |  |  |  |  |  |  |
|  |  |  |  |  |  |  |  |
|  |  |  |  |  |  |  |  |
|  |  |  |  |  |  |  |  |
|  |  |  |  |  |  |  |  |
|  |  |  |  |  |  |  |  |
|  |  |  |  |  |  |  |  |
