# Supplementary material for: Translation and cultural adaptation of the CLEFT-Q for use in Colombia, Chile, and Spain
Source: Health Qual Life Outcomes. 2017 Nov 28;15:228. doi: 10.1186/s12955-017-0805-7 (PMC5704495; doi:10.1186/s12955-017-0805-7)
Supplement: Supplementary file 4 — Template data collection and analysis form for cognitive debriefing interviews. (DOCX 65 kb) [file 12955_2017_805_MOESM4_ESM.docx]

**Supplementary file 4.** Template data collection and analysis form for cognitive debriefing interviews

| Study ID | Age | Gender | Cleft type | CLEFT-Q scale | CLEFT-Q item | What was the difficulty? | What was the suggested change? |
| --- | --- | --- | --- | --- | --- | --- | --- |
|  |  |  |  |  |  |  |  |
|  |  |  |  |  |  |  |  |
|  |  |  |  |  |  |  |  |
|  |  |  |  |  |  |  |  |
|  |  |  |  |  |  |  |  |
|  |  |  |  |  |  |  |  |
|  |  |  |  |  |  |  |  |
|  |  |  |  |  |  |  |  |
|  |  |  |  |  |  |  |  |
|  |  |  |  |  |  |  |  |
|  |  |  |  |  |  |  |  |
|  |  |  |  |  |  |  |  |
|  |  |  |  |  |  |  |  |
|  |  |  |  |  |  |  |  |
|  |  |  |  |  |  |  |  |
|  |  |  |  |  |  |  |  |
